# Low-frequency electroacupuncture attenuates methamphetamine-induced depressive-like behaviors and cognitive impairment via modulating neuroinflammation

**DOI:** 10.3389/fneur.2025.1652065

**Published:** 2025-10-20

**Authors:** Jingyi Zhang, Rongji Hui, Jiabao Xu, Ludi Zhang, Bing Xie, Chunling Ma, Yi Li, Yueli Zou, Di Wen, Xiujun Yu

**Affiliations:** ^1^Key Laboratory of Clinical Neurology, Ministry of Education, Hebei Medical University, Department of Neurology, the Second Hospital of Hebei Medical University, Key Neurological Laboratory of Hebei Province, Shijiazhuang, Hebei, China; ^2^Hebei Key Laboratory of Forensic Medicine, Collaborative Innovation Center of Forensic Medical Molecular Identification, Research Unit of Digestive Tract Microecosystem Pharmacology and Toxicology, Chinese Academy of Medical Sciences, College of Forensic Medicine, Hebei Medical University, Shijiazhuang, Hebei, China; ^3^Hebei Medical University Basic Medicine Postdoctoral Research Station, Shijiazhuang, Hebei, China

**Keywords:** methamphetamine, depressive-like behaviors, cognitive impairment, electroacupuncture, neuroinflammation

## Abstract

**Introduction:**

Methamphetamine (METH) abuse primarily affects the central nervous system (CNS), leading to CNS damage and contributing to depressive-like behaviors, cognitive impairment, and other neuropsychiatric disorders. Electroacupuncture (EA) has shown promise in treating mental disorders linked to CNS damage, yet the effects of EA on METH-induced depressive-like behaviors and cognitive impairment and it’s underlying therapeutic mechanisms remain largely unclear.

**Methods:**

In this study, a mouse model of METH-induced neuropsychiatric dysfunction was established by administering high-dose METH under elevated ambient temperature. EA was applied at different frequencies to the Zusanli (ST36) acupoint for 7 days post-METH administration.

**Results:**

Behavioral tests revealed that low-frequency EA significantly alleviated depressive-like behaviors and cognitive impairment. Additionally, EA restored blood-brain barrier (BBB) integrity, as evidenced by Western blotting (WB) and Evans blue staining. Neuronal injury was attenuated, as shown by Nissl and hematoxylin and eosin (HE) staining. Further investigations into neuroinflammation revealed that EA suppressed microglial activation in the hippocampus, decreased the expression of IL-6 and TNF-*α*, and inhibited the NF-κB/NLRP3 signaling pathway.

**Discussion:**

The present study suggested that EA alleviates METH-induced depressive-like behaviors and cognitive impairment by modulating neuroinflammation, particularly through the inhibition of microglial activation and pro-inflammatory cytokine release. EA may represent a promising non-pharmacological strategy for the treatment of METH-associated neuropsychiatric disorders.

## 1 Introduction

Drug abuse remains a critical global public health and security concern (1). Methamphetamine (METH) ranks among the most extensively abused psychostimulants worldwide (2), which is particular concern due to its high addictive potential and severe neurotoxic and neuropsychiatric effects (3). High-dose or long-term METH use frequently leads to a range of psychiatric disorders, including emotional dysregulation, cognitive impairment, and psychotic symptoms such as anxiety, depression, paranoia, delirium and mania (3–6). Currently, effective pharmacological interventions for METH addiction remain largely unavailable, highlighting the urgent need to explore novel therapeutic strategies and underlying mechanisms associated with METH-induced neurological and neuropsychiatric disorders.

The METH abuse is associated with complex and multifaceted neurotoxic effects (7). Current evidence suggests that the primary mechanisms of METH-induced neuropsychiatric dysfunction involve a complex interplay of pathophysiological alterations, including hyperthermia, systemic and neuroinflammatory responses, oxidative stress, excitotoxicity due to amino acid imbalances, and mitochondrial dysfunction (8, 9). These disturbances disrupt neurotransmitter homeostasis, impair synaptic plasticity (10), and eventually lead to widespread neural circuit disability and behavioral abnormalities (11). Among these mechanisms, neuroinflammation has emerged as a central and critical contributor to METH-induced neuropathology (12). Mounting evidence indicates that METH triggers a robust inflammatory response in central nervous system (CNS) (13, 14). This inflammation is increasingly recognized as a driving factor in the development of depressive-like behaviors and cognitive impairment caused by METH abuse. Therefore, modulating neuroinflammation may represent a promising therapeutic approach to mitigate the METH exposure-associated depressive-like behaviors and cognitive impairment.

Acupuncture is a fundamental therapeutic modality in traditional Chinese medicine. Electroacupuncture (EA), which integrates acupuncture with modern electrical stimulation techniques, delivers low-frequency currents to specific acupoints to modulate physiological functions (15, 16). EA has demonstrated therapeutic potential in various neuropsychiatric disorders, including cognitive impairment and depressive-like behaviors (17). Notably, accumulating evidence highlights the anti-inflammatory and immune-regulatory effects of EA (18–21). For instance, EA has been found to improve cognitive impairment and inflammation in models of cerebral ischemia–reperfusion injury (22, 23). Furthermore, EA intervention of the Zusanli (ST36) acupoint has demonstrated enhanced immune function in post-surgical animals and exert anti-inflammatory effects (24). However, there remains a significant gap in understanding whether EA stimulation at ST36 acupoint can attenuate METH-induced depressive-like behaviors and cognitive impairment.

Herein, we employed the open field test (OFT), tail suspension test (TST), forced swimming test (FST), and novel object recognition test (NORT) to evaluate the efficacy of EA in alleviating METH-induced depressive-like behaviors and cognitive impairment. In addition, we assessed blood–brain barrier (BBB) integrity, neuronal damage and evaluated neuroinflammation by measuring the expression of IL-6, TNF-α, nuclear factor kappa-B (NF-κB), NLRP3 in hippocampus. The study aims to elucidate the neurobiological mechanisms underlying the therapeutic effects of EA on METH-induced depressive-like behaviors and cognitive impairment, with a focus on its role in regulating hippocampal inflammation.

## 2 Materials and methods

### 2.1 Animals

Male C57BL/6N mice (6–8 weeks old) were procured from Beijing Vital River Laboratory Animal Technology Co., Ltd. and housed under specific pathogen-free (SPF) conditions at the Experimental Animal Center of Hebei Medical University. Animals were maintained in a controlled environment with a temperature of 22 °C, relative humidity of ~60%, and a 12-h light/dark cycle (lights off from 7:00 to 19:00), with a maximum of five mice per cage. All experimental procedures involving animals were conducted in accordance with ethical guidelines and approved by the Institutional Animal Care and Use Committee of Hebei Medical University (Approval No.: IACUC-Hebmu-202023).

### 2.2 Experimental design and intervention

Building upon our previous research, a mouse model of METH-induced depressive-like behaviors and cognitive impairment was established. Mice were then randomly assigned to five experimental groups:

Saline group: Mice were administered intraperitoneal (i.p.) injections of saline.

Saline + Anesthetic group: Mice were administered (i.p.) injections of saline and, during EA sessions, were administered 2% pentobarbital sodium (i.p.) to control for anesthetic effects.

METH + Anesthetic group: Mice were administered METH (10 mg/kg, i.p.) four times at 2-h intervals at 28 °C. During EA sessions, they were also given 2% pentobarbital sodium to eliminate the influence of anesthesia.

METH + EA 10 Hz group (High-frequency EA): Mice were administered METH injections as above and underwent EA at the ST36 acupoints twice daily for 7 consecutive days (30 min per session, 6-h interval), using 10 Hz frequency, 1 mA current, and 0.25 × 13 mm needles.

METH + EA 2 Hz group (Low-frequency EA): This group were administered identical METH treatment and EA procedure as the 10 Hz group, but with a 2 Hz stimulation frequency.

This acute binge-exposure paradigm was selected to model the scenario of recreational binge use leading to acute neurotoxicity, which allows for the efficient investigation of early pathological events and therapeutic interventions.

### 2.3 Open field test (OFT)

At the onset of the test, each mouse was positioned in the center area of the arena. Following a 5-min acclimatization period, the mouse’s movement trajectories were recorded using the Noldus EthoVision XT video tracking software (Wageningen, Netherlands). Total distance traveled and time spent in the central zone were recorded to evaluate anxiety-related behaviors.

### 2.4 Tail suspension test (TST)

Each mouse was suspended by the tail with head facing downward and prevented any contact with the desktop or surrounding walls. After a 1-min acclimatization period, the immobility time of the mouse was recorded over a total measurement duration of 5 min.

### 2.5 Forced swimming test (FST)

Mice were positioned in a cylindrical transparent container filled with water (bucket diameter: 20 cm, height: 40 cm), with the temperature of the water controlled between 23 and 25 °C and the level of the water set at 17 cm. After gently placing the mice into the water, a 1-min acclimatization period was allowed. Subsequently, the immobility time, reflecting a state of despair behavior, was recorded over a total duration of 5 min. Immobility was defined as the absence of active movement of the hind limbs.

### 2.6 Novel object recognition test (NORT)

Cognitive abilities in mice were assessed using the NORT. On the first day of the experiment, mice were positioned in an empty arena to explore freely, once in the morning and once in the afternoon, with each session lasting 10 min. On the second day, two identical familiar objects (A, A) were positioned in symmetric positions on one side of the testing box, with both objects positioned 10 centimeters away from the side walls and the back wall. Mice were gently placed in the center of the opposite wall and allowed to explore the experimental arena freely for a duration of 5 min. During this time, the number of times the mice’s nose or mouth came into contact with the familiar objects (A, A) and the exploration time within 2–3 centimeters of the objects were recorded. After 4 h, a short-term memory and learning test was conducted by replacing one of the familiar objects (A) with a novel object (B). After 24 h, the novel object (B) was replaced with another novel object (C), and the experiment was repeated. The exploration time for the novel objects (B, C) and the familiar object (A) was recorded. The Discrimination Index was used as the statistical measure and calculated using the formula N/(N + F) × 100%, where N represents the exploration time for the novel objects (B, C) and F represents the exploration time for the familiar object (A).

### 2.7 Nissl staining

Mouse brain tissue sections were stained with Methyl Blue (G1434, Solarbio Life Science, Beijing, China) for 10 min and differentiated in Nissl staining buffer. Subsequently, the sections were treated with ammonium molybdate for 3 min, followed by sequential dehydration using 70% alcohol, 80% alcohol, 95% alcohol, anhydrous ethanol, and xylene. After dehydration, the sections were immediately rinsed with distilled water. The sections were then mounted with neutral resin and examined under a microscope to assess pathological morphology in the hippocampal region of the mice. The area of Nissl bodies was quantified using ImageJ software (Media Cybernetics, United States).

### 2.8 Evans blue staining

Mice were injected with Evans blue staining solution at a ratio of 2–3 mL/kg. Within 10 s after injection, the eyes and tails of the mice gradually turned blue, and within 1 h, the paws and ears also exhibited a blue coloration. After 1 h, the mice were sacrificed, and their brain tissues were harvested. The tissues were fixed in 4% paraformaldehyde for 48 h. Subsequently, the hippocampal region was excised, dehydrated, embedded in paraffin, and sectioned into 5 μm-thick slices, which were mounted onto glass slides. The sections were then dewaxed and subjected to antigen retrieval. A specific primary antibody, Rabbit Anti-CD31 pAb (1:200, GB11063-2, Servicebio, Wuhan, China), was applied for incubation. This was followed by incubation with a goat anti-rabbit fluorescent dye-conjugated secondary antibody (1:2000, A21207, Invitrogen, United States). Cell nuclei were stained with DAPI (8961S, Cell Signaling Technology, United States). After completion of the staining process, the sections were observed under a fluorescence confocal laser microscope (Leica, Germany), and the data were analyzed using ImageJ software.

### 2.9 Hematoxylin and eosin (HE) staining

Mice were euthanized, and their brain tissues were harvested, fixed in 10% formaldehyde, embedded in paraffin, and sectioned at a thickness of 5 μm. The paraffin-embedded sections were deparaffinized with xylene and rehydrated through a graded ethanol series (100, 95, and 80%, each for 5 min). Subsequently, the sections were stained with Harris hematoxylin for 1 min, differentiated with 1% hydrochloric acid ethanol for 3 s, rinsed in distilled water, and dehydrated using a graded ethanol series. Eosin staining was performed for 40 s. Final dehydration was completed with absolute ethanol and xylene, and the sections were mounted using neutral resin. Histological images were captured using a Leica Aperio CS2 microscope (Leica, Germany).

### 2.10 Immunofluorescence (IF) assay

Mouse brain tissues were fixed in 4% paraformaldehyde for 48 h. The hippocampal regions were dissected, dehydrated through a graded alcohol series (75, 85, and 95%, absolute ethanol), cleared in xylene, and embedded in paraffin. Serial sections (5 μm thick) were prepared and mounted onto glass slides, followed by xylene treatment for 10 min to remove residual paraffin. After antigen retrieval, the sections were incubated overnight at 4 °C with specific primary antibodies: anti-Iba-1 (1:100, ET1705-78, Huaan, Zhejiang, China) and anti-NeuN (1:200, HA601111, Huaan, Zhejiang, China). Following PBS washes, a goat anti-rabbit fluorescent secondary antibody (1:2000, A21207, Invitrogen, United States) was applied for 5 min, then washed six times with PBS (5 min per wash). Nuclei were counterstained with DAPI (8961S, Cell Signaling Technology, United States). Fluorescent images were obtained using a confocal laser scanning microscope (Leica, Germany), and image analysis was performed using ImageJ software.

### 2.11 Western blotting (WB) analysis

Mouse brain tissues were collected, and the hippocampal region was added to RIPA lysis buffer and homogenized thoroughly. The homogenate was centrifuged at 4 °C, and the supernatant was collected. Protein concentration was determined using the BCA method. Equal amounts of protein samples were denatured and subjected to electrophoresis on a 10% SDS-PAGE gel. Following separation, the proteins were electrophoretically transferred onto a 0.45 μm PVDF membrane (Immobilon^®^-P, IPVH0010, Merck Millipore, United States). This membrane was then incubated for 2 h at ambient temperature in a blocking solution consisting of Tris-buffered saline fortified with 5% skim milk and 0.1% Tween-20 (TBST). After blocking, the membrane was incubated with designated primary antibodies at 4 °C overnight, including Mouse Anti-Claudin-5 Antibody (1:1000, 35–2,500, Invitrogen, United States), Rabbit Anti-Occludin Antibody (1:1000, R1510-33, Huaan, China), Rabbit Anti-IL-6 Antibody (1:1000, DF6087, Affinity Biosciences, USA), Rabbit Anti-TNF-Alpha Antibody (1:1000, AF7014, Affinity Biosciences, USA), Rabbit Anti-NF-κB p65 Antibody (1:1000, D14E12, Cell Signaling Technology, United States), Rabbit Anti-NLRP3 Antibody (1:1000, ET1610-93, Huaan, Zhejiang, China), diluted together with Rabbit Anti-GAPDH Antibody (1:10000, ET1601-4, Huaan, China) or Rabbit Anti-beta Tubulin Antibody (1:80000, ab179513, Abcam, United Kingdom). The membrane was washed four times with TBST, 5 min each time, and then incubated with goat anti-rabbit secondary antibodies (1:5000, D40416-05, Licor, United States) or goat anti-mouse secondary antibodies (1:5000, D40409-05, Licor, USA) in a 37 °C constant-temperature shaker for 1 h in the dark. Following incubation, the membrane was rinsed three times with TBST (5 min each), followed by a final wash with TBS for 5 min. Protein signals were visualized using the Odyssey imaging system, and band intensities were quantified with ImageJ software. Statistical analyses were conducted using GraphPad Prism version 8.4.3.

### 2.12 Statistical analysis

In this study, we utilized GraphPad Prism 8.4.3 for our statistical analyses. All results were presented as the mean with standard error of the mean (SEM). We evaluated group variances through a one-way analysis of variance (ANOVA). To delve deeper, we employed the Bonferroni correction for post-hoc analysis. A threshold of *p* < 0.05 was set to denote statistical significance.

## 3 Results

### 3.1 Low-frequency EA alleviates METH-induced anxiety and depressive-like behaviors

**Figure 1:**
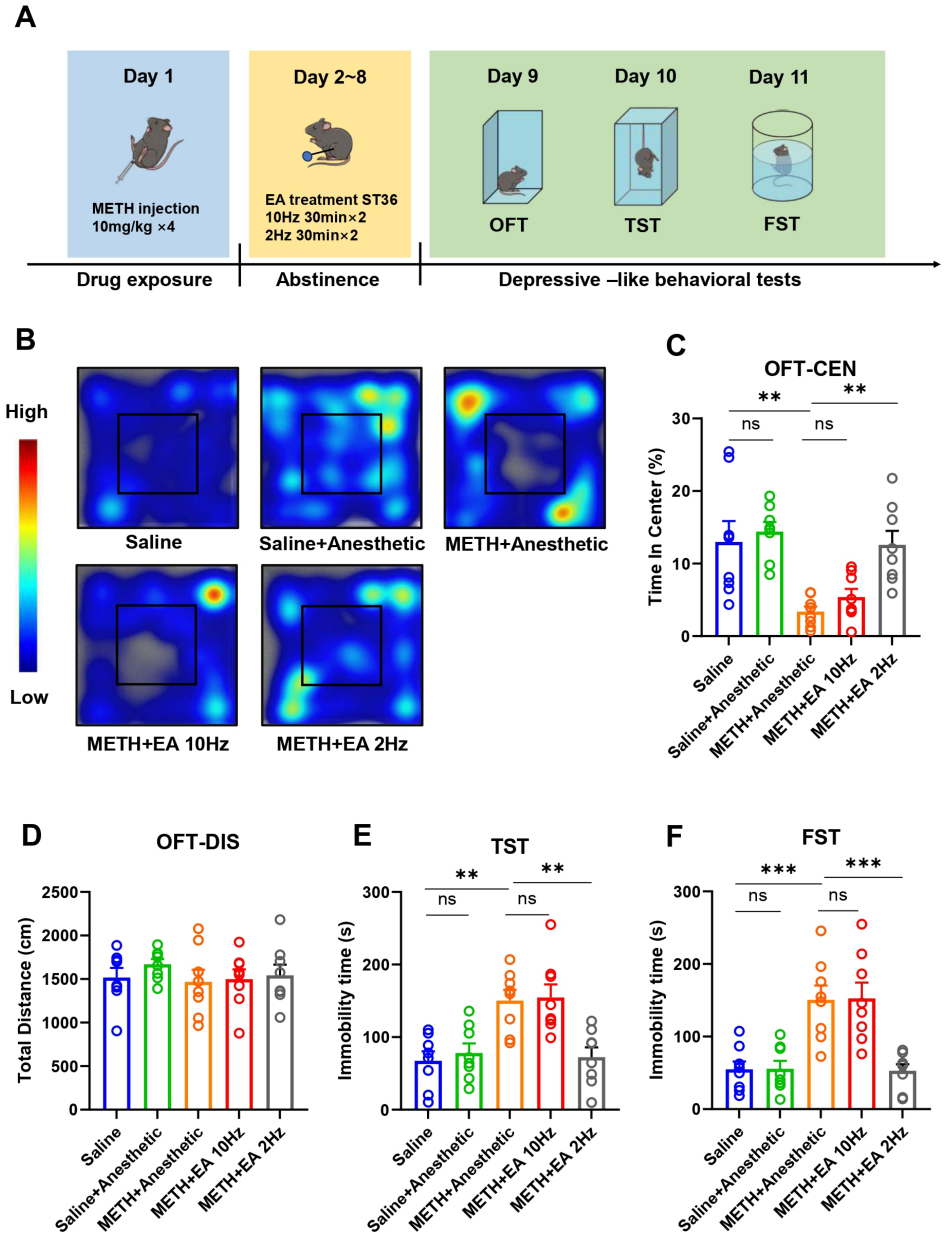
Effect of EA on anxiety and depressive-like behaviors in mice exposed to METH. **(A)** Timeline of drug administration, EA intervention, and behavioral test. **(B)** The movement trajectories in heatmap. **(C)** Time in center in OFT. **(D)** Total distance in OFT. **(E)** The duration of immobility observed in mice during TST. **(F)** The duration of immobility observed in mice during FST. **p < 0.01 and ***p < 0.001 compared with Saline group; **p < 0.01 and ***p < 0.001 compared with METH+Anesthetic group. *n* = 8 in each group.

To establish a model of METH-induced depressive-like behaviors, C57BL/6 N mice (7–8 weeks old, 20–25 g) received four intraperitoneal injections of METH (10 mg/kg) at 2-h intervals under ambient temperature (28 °C). Control mice received an equivalent volume of saline. Following METH administration, EA was applied at the ST36 acupoints at different frequencies for 7 consecutive days, and behavioral tests were conducted at the end of treatment. The timeline of drug administration, EA intervention and behavioral tests is depicted in Figure 1A.

In the OFT, no significant difference in total distance traveled was observed between groups [*F*_(4.000, 29.92)_ = 0.4817, *p* = 0.7489, *η*^2^ = 0.063 (negligible), *f* = 0.26 (small), Cohen’s *d* < 0.2; Figure 1D], indicating no change in overall locomotor activity. However, the percentage of time spent in the central zone, indicative of anxiety- and depressive-like behaviors, differed significantly among groups [*F*_(4.000, 19.27)_ = 8.054, *p* = 0.0005, *η*^2^ = 0.626 (large), *f* = 1.29 (large), Cohen’s *d* = 1.1–2.0; Figures 1B,C]. *Post hoc* analysis showed that METH administration significantly reduced central zone time compared to saline (*p* = 0.0001), confirming the establishment of depressive-like behaviors. Low-frequency EA significantly increased time spent in the center compared to the METH group (*p* = 0.0107), whereas high-frequency EA had no significant effect (*p* = 0.5814). Anesthetics alone did not affect behavior (*p* = 0.9900).

In the TST, immobility time significantly differed among groups [*F*_(4.000, 32.58)_ = 8.985, *p* < 0.0001, *η*^2^ = 0.525 (large), *f* = 1.05 (large), Cohen’s *d* = 0.9–1.7; Figure 1E]. METH exposure significantly increased immobility compared to saline (*p* = 0.0194), while low-frequency EA significantly reduced immobility (*p* = 0.0121). No significant improvement was observed with high-frequency EA (*p* = 0.9997).

Similarly, in the FST, significant group differences in immobility time were observed [*F*_(4.000, 23.95)_ = 12.00, *p* < 0.0001, *η*^2^ = 0.667 (large), *f* = 1.41 (large), Cohen’s *d* = 1.2–2.1; Figure 1F]. METH increased immobility (*p* = 0.0106), which was reversed by low-frequency EA (*p* = 0.0084), but not by high-frequency EA (*p* > 0.9999).

The experiments described above demonstrate that low-frequency EA treatment ameliorated the METH-induced depressive- and anxiety-like behavior. Notably, high-frequency EA did not alter these behaviors.

### 3.2 Low-frequency EA mitigates METH-induced impairment of memory functions

**Figure 2:**
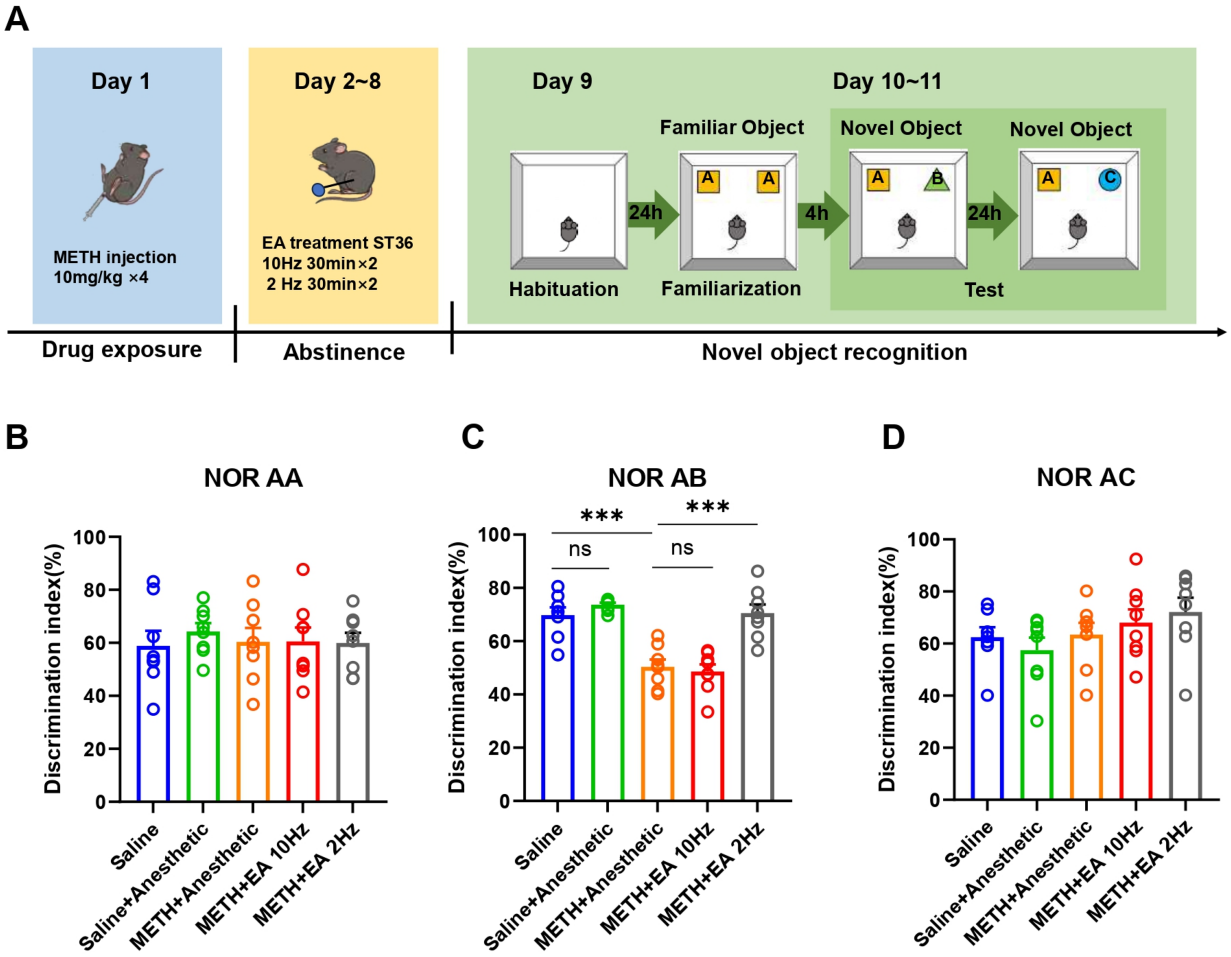
Effect of EA on cognition function in mice exposed to METH. **(A)** Timeline of EA interventions after drug administration and NORT. **(B)** The discrimination index of mice in **(A, A)**. **(C)** The discrimination index of mice in **(A, B)**. **(D)** The discrimination index of mice in **(A, C)**. ***p < 0.001 compared with Saline group and METH+Anesthetic group. *n* = 8 in each group.

To assess cognitive function, the NORT was conducted (Figure 2A). During the familiarization phase (two identical objects), no significant group differences were detected [*F*_(4.000, 30.57)_ = 0.1868, *p* = 0.9435, *η*^2^ = 0.024 (negligible), *f* = 0.16 (small), Cohen’s *d* < 0.2; Figure 2B]. Four hours later, one object was replaced with a novel one to assess short-term memory function. Recognition index significantly differed among groups [*F*_(4.000, 28.16)_ = 20.84, *p* < 0.0001, *η*^2^ = 0.748 (large), *f* = 1.72 (large), Cohen’s *d* = 1.6–2.5; Figure 2C]. METH significantly reduced the recognition index compared to saline (*p* = 0.0002), while low-frequency EA significantly improved it (*p* = 0.0030). No improvement was seen with high-frequency EA (*p* = 0.9894). No significant differences were observed 24 h later in the second recognition test [*F*_(4.000, 33.20)_ = 1.387, *p* = 0.2598, *η*^2^ = 0.144 (small), *f* = 0.41 (small), Cohen’s *d* = 0.2–0.5; Figure 2D]. This test evaluates long-term memory retention.

### 3.3 Low-frequency EA protects against METH-induced BBB disruption in the hippocampus

**Figure 3:**
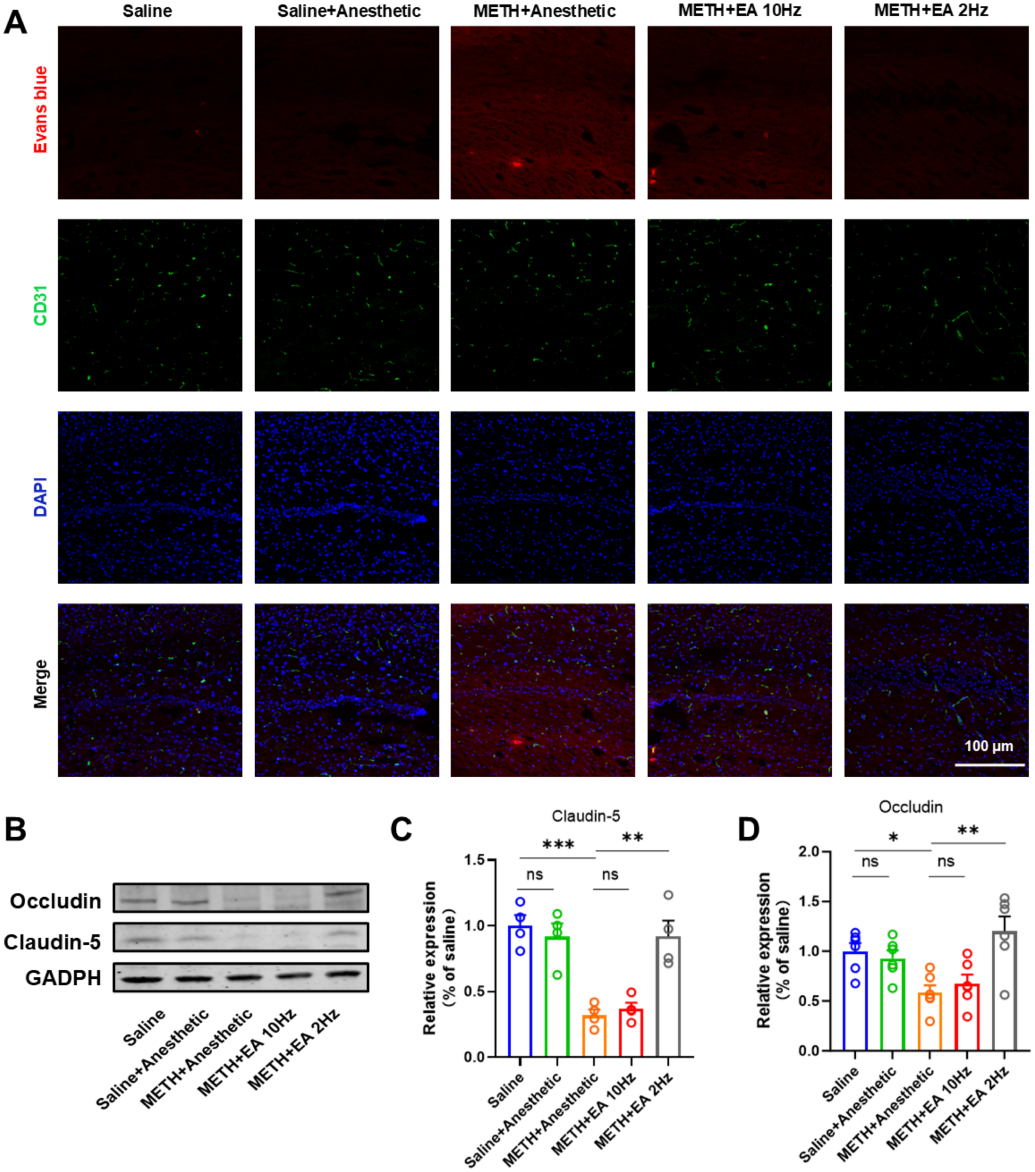
Effect of EA on BBB permeability changes in hippocampal CA1 region of mice exposed to METH. **(A)** Infiltration of Evans Blue in Hippocampus CA1. **(B)** Representative blots showing Occludin and Claudin-5 expression. **(C,D)** Relative amounts quantified by densitometric quantification of changes in gray values. *p < 0.05 and ***p < 0.001 compared with Saline group; **p < 0.01 compared with METH+Anesthetic group. *n* = 6 in each group.

Evans blue dye injection revealed increased BBB permeability in the hippocampal CA1 region of METH-treated mice. Blood vessels were visualized by immunofluorescent staining as described above using a CD31 (PECAM-1) specific antibody. IF staining showed extensive dye leakage in the METH group but not in the saline or METH+EA 2 Hz groups. Dye infiltration was still present in the METH+EA 10 Hz group (Figure 3A).

WB analysis confirmed decreased expression of tight junction proteins Occludin and Claudin-5 in the METH group (Figure 3B) (Claudin-5: *p* = 0.0239, Figure 3C; Occludin: *p* = 0.0263, Figure 3D), which was significantly restored by low-frequency EA (Claudin-5: *p* = 0.0448; Occludin: *p* = 0.0399). No significant recovery was observed in the high-frequency EA group (Claudin-5: *p* = 0.9370; Occludin: *p* = 0.9367) [Claudin-5: *F*_(4.000, 10.37)_ = 15.63, *p* = 0.0002, *η*^2^ = 0.857 (large), *f* = 2.46 (large), Cohen’s *d* = 1.9–3.1; Figure 3C] [Occludin: *F*_(4.000, 17.90)_ = 6.34, *p* = 0.0023, *η*^2^ = 0.586 (large), *f* = 1.19 (large), Cohen’s *d* = 1.0–1.8]. Anesthetics alone did not alter protein expression.

### 3.4 Low-frequency EA attenuates METH-induced neuronal damage in the hippocampal CA1 region

**Figure 4:**
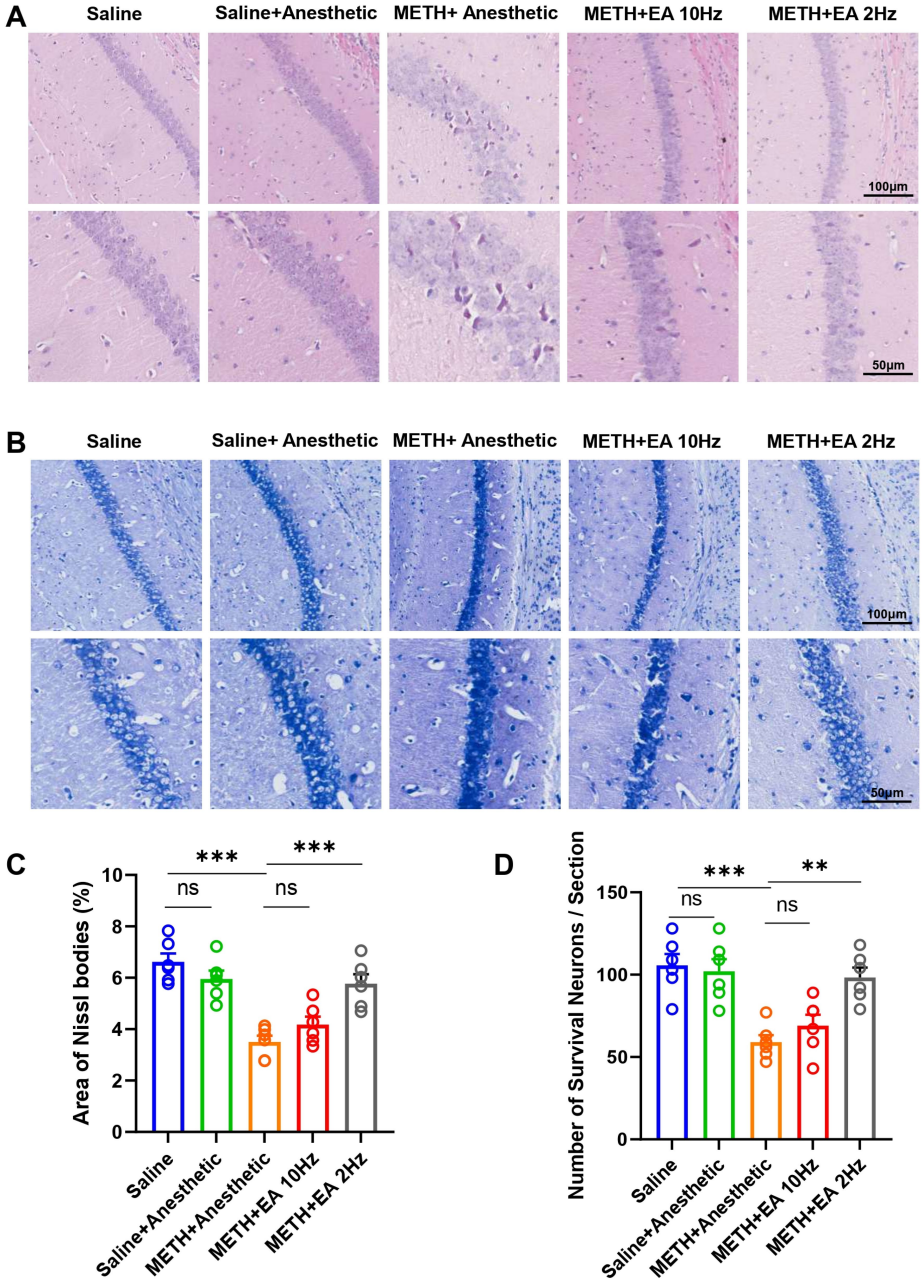
Effect of EA on neuron damage in hippocampal CA1 region of mice exposed to METH. **(A)** Representative microphotographs of HE Staining in the hippocampal CA1 region. **(B)** Representative microphotographs of Nissl Staining in the CA1 region. **(C)** The area of Nissl bodies. **(D)** The number of Nissl positive neurons per field of view. ***p < 0.001 compared with Saline group; **p < 0.01 and ***p < 0.001 compared with METH+Anesthetic group. *n* = 6 in each group.

To investigate the impact of EA on METH-induced histopathological changes in the hippocampal CA1 region, HE staining was conducted on adjacent tissue sections. HE staining revealed substantial neuronal damage in the CA1 region following METH treatment, including nuclear condensation and disorganization. And eosinophilic cytoplasm with bland, uniform nuclear features. These pathological changes were markedly improved by low-frequency EA, while high-frequency EA showed limited effect (Figure 4A).

Subsequently, we performed Nissl staining on the sections to visualize the morphological characteristics of neurons and assess positive neuronal loss (Figure 4B). The Nissl staining results were consistent with those of the HE staining. Nissl staining analysis revealed a significant reduction in Nissl body area (*p* = 0.0011, Figure 4C) [*F*_(4.000, 23.63)_ = 17.12, *p* < 0.0001, *η*^2^ = 0.744 (large), *f* = 1.70 (large), Cohen’s *d* = 1.5–2.4; Figure 4C] and positive neuron number (*p* = 0.0070, Figure 4D) [*F*_(4.000, 22.67)_ = 11.25, *p* < 0.0001, *η*^2^ = 0.665 (large), *f* = 1.41 (large), Cohen’s *d* = 1.2–2.1; Figure 4D] in METH-treated mice. The Nissl body area was significantly restored by low-frequency EA (*p* = 0.0044), but not by high-frequency EA (*p* = 0.4661). Similarly, the positive neuron number exhibited analogous outcomes (Low-frequency EA: *p* = 0.0029; high-frequency EA: *p* = 0.7191).

### 3.5 Low-frequency EA suppresses METH-induced hippocampal microglia activation and inflammatory signaling pathway

**Figure 5:**
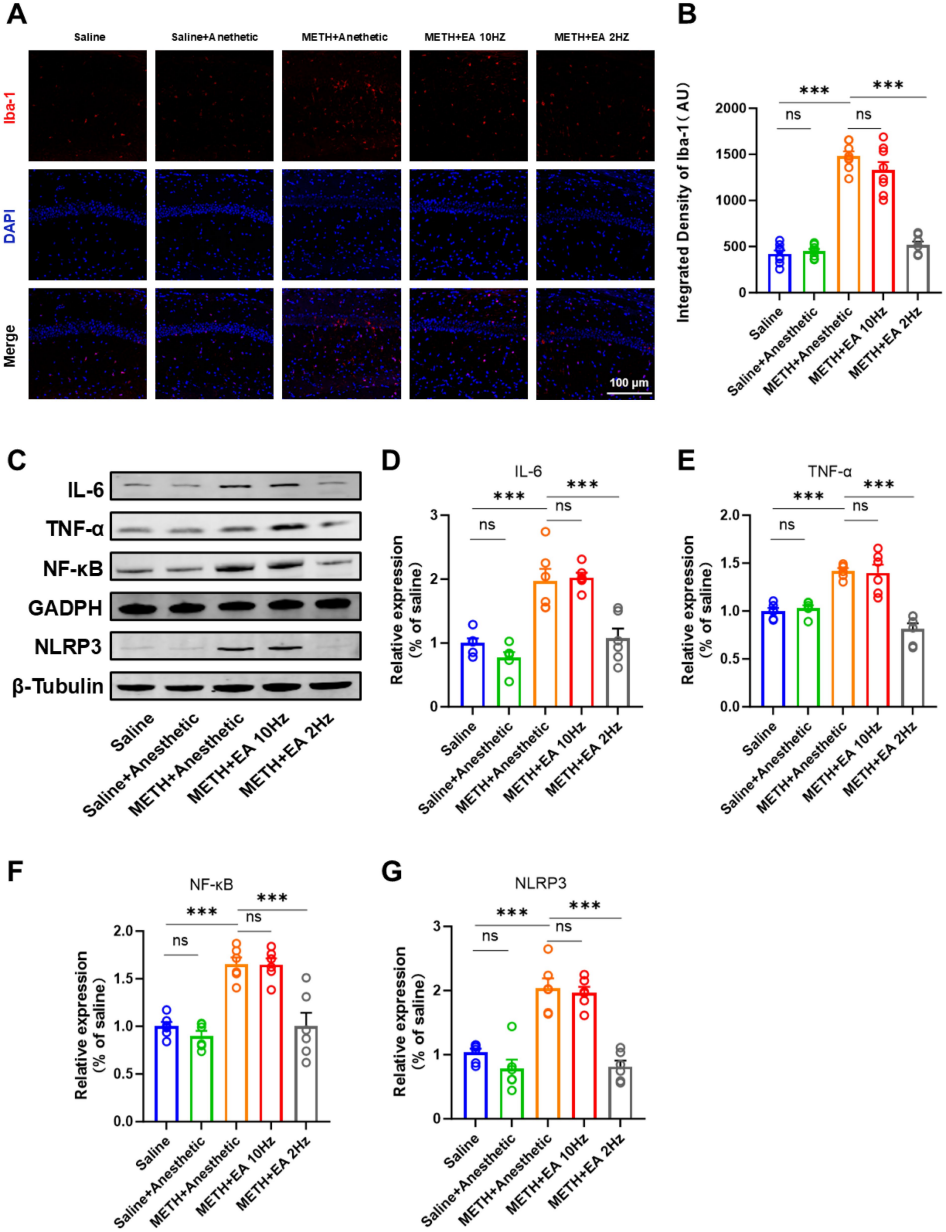
Effect of EA on the level of neuroinflammation in mice exposed to METH. **(A,B)** Relative fluorescence intensity of Iba-1 in representative IF staining. **(C)** Representative blots of the NF-κB/NLRP3 signaling pathway showing protein expression (Relative amounts quantified by densitometric quantification of changes in gray values). **(D)** Relative expression changes in protein imprinting of IL-6. **(E)** Relative expression changes in protein imprinting of TNF-α. **(F)** Relative expression changes in protein imprinting of NF-κB. **(G)** Relative expression changes in protein imprinting of NLRP3. ***p < 0.001 compared with Saline group; ***p < 0.001 compared with METH+Anesthetic group. *n* = 6 in each group.

IF staining showed activated microglia with amoeboid morphology and an enlarged soma with few blunt or no processes in the hippocampal CA1 region of METH-treated mice. Low-frequency EA reduced microglial activation, while high-frequency EA did not (Figure 5A). Meanwhile, we utilized the microglial marker Iba-1 to evaluate microglial activation. Quantification of Iba-1 fluorescence intensity confirmed these findings [*F*_(4.000, 18.26)_ = 99.06, *p* < 0.0001, *η*^2^ = 0.956 (large), *f* = 4.63 (large), Cohen’s *d* = 3.5–5.2; Figure 5B]. Low-frequency EA significantly reduced Iba-1 expression (*p* < 0.0001), but high-frequency EA showed no effect (*p* = 0.5882).

To elucidate the potential mechanism underlying the therapeutic effects of EA against METH-induced neurotoxicity, we investigated the NF-κB/NLRP3 signaling pathway, a key mediator implicated in CNS neuroinflammation. WB analysis revealed significantly elevated levels of IL-6, TNF-*α*, NF-κB, and NLRP3 in the METH group (Figure 5C), which were attenuated by low-frequency EA (IL-6: *p* = 0.0328; TNF-α: *p* = 0.0002; NF-κB: *p* = 0.0228; NLRP3: *p* = 0.0009). High-frequency EA did not significantly alter these protein levels (IL-6: *p* = 0.9980; TNF-α: *p* = 0.9990; NF-κB: *p* > 0.9999; NLRP3: *p* = 0.9942; Figures 5D–G) [IL-6: *F*_(4.000, 15.06)_ = 21.45, *p* < 0.0001, *η*^2^ = 0.850 (large), *f* = 2.38 (large), Cohen’s *d* = 2.0–3.2; Figure 5D] [TNF-α: *F*_(4.000, 13.40)_ = 25.46, *p* < 0.0001, *η*^2^ = 0.883 (large), *f* = 2.75 (large), Cohen’s *d* = 2.3–3.5; Figure 5E] [NF-κB: *F*_(4.000, 13.00)_ = 21.02, *p* < 0.0001, *η*^2^ = 0.866 (large), *f* = 2.55 (large), Cohen’s *d* = 2.1–3.3] [NLRP3: *F*_(4.000, 18.41)_ = 29.99, *p* < 0.0001, *η*^2^ = 0.867 (large), *f* = 2.56 (large), Cohen’s *d* = 2.2–3.4].

## 4 Discussion

This study demonstrated that low-frequency (2 Hz) EA at the ST36 acupoint significantly ameliorates METH-induced depressive-like behaviors and cognitive impairment. This effect was associated with the restoration of BBB integrity, attenuation of hippocampal neuronal damage, and inhibition of neuroinflammatory responses mediated by microglial activation and the NF-κB/NLRP3 signaling pathway. Notably, high-frequency EA (10 Hz) did not produce comparable protective effects. Therefore, our findings indicated that EA served as a promising therapeutic approach for decreasing METH-induced depressive-like behaviors and cognitive impairment.

METH administration is associated with a broad range of neuropsychiatric behaviors in mice, including cognitive impairment and depressive-like behaviors (25, 26). In this study, high-dose METH exposure resulted in increased immobility time in the TST and FST, as well as reduced exploratory behavior in the OFT, suggesting the successful induction of anxiety and depressive-like behaviors and cognitive impairment. In the NORT, METH-induced mice displayed a significantly lower recognition index, indicating impaired recognition memory. Consistent with the behavioral phenotypes reported in our previously established binge-dose paradigm, the present data confirm the robust reproducibility and experimental stability of this model (27, 28). Importantly, single-binge administration does not recapitulate the progressive neuroadaptations that define chronic METH addiction; sustained-exposure paradigms are required to model the persistent mnemonic and executive deficits observed in long-term users. The present observation of transient short-term memory impairment with intact long-term memory, however, aligns with acute binge exposure rather than chronic regimens. This high-dose paradigm replicates a clinically relevant scenario in which emergency care is sought after recreational binge use, endowing the binge model with unique translational value for early intervention and prevention strategies against stimulant abuse. Beyond their divergent translational contexts, chronic-exposure paradigms are anticipated to diverge from acute binge-dose regimens in progressive neuroadaptive trajectories, sustained mnemonic and executive deficits, and the underlying molecular cascades; elucidating these distinctions is the focal axis of our forthcoming investigations. Therefore, the findings of the present study are primarily applicable to elucidating intervention mechanisms underlying acute METH toxicity; their therapeutic relevance in chronic users remains to be prospectively validated. In subsequent studies, we plan to focus particularly on the more complex mechanisms underlying chronic METH exposure, including neural plasticity alterations, sustained glial cell activation, and epigenetic regulation. These investigations will be integrated with the NF-κB/NLRP3 inflammatory pathway identified in the current study, aiming to comprehensively evaluate the therapeutic potential of EA in treating METH-related neuropsychiatric disorders.

Notably, the results indicated that low-frequency EA stimulation significantly alleviated anxiety, cognitive impairment and depressive-like behaviors in METH-induced mice, whereas high-frequency EA did not produce such effects. These findings are consistent with prior reports indicating that EA can modulate emotional and cognitive behaviors by influencing hippocampal and prefrontal neural circuits (29–31). However, few studies have investigated this approach in the context of METH-induced neuropsychiatric dysfunction, thus emphasizing the novelty of our findings.

Mechanistically, our results show that low-frequency EA preserves the structural and functional integrity of the BBB in the hippocampal CA1 region. METH administration significantly disrupted BBB permeability, as evidenced by elevated Evans blue extravasation and downregulation of Occludin and Claudin-5. Our findings revealed that low-frequency EA intervention significantly mitigated these changes, whereas not by high-frequency EA. The BBB plays a crucial role in maintaining CNS homeostasis, and its disruption is associated with increased vulnerability to peripheral immune cell infiltration and neuroinflammation (32–35). Therefore, the observed BBB-protective effect of EA may represent a key mechanism underlying its therapeutic potential.

In addition to BBB damage, METH exposure led to extensive neuronal injury in the hippocampal CA1 region, including disorganized cell arrangement, nuclear fragmentation, and loss of Nissl substance. These histopathological changes were accompanied by a significant reduction in the number of surviving neurons. However, low-frequency EA was able to attenuate these morphological changes and neuronal damage, suggesting a neuroprotective effect that may contribute to the observed improvements in depressive-like behaviors and cognitive impairment. These findings align with previous reports demonstrating that EA can reduce neuronal apoptosis and promote synaptic plasticity under pathological conditions (36, 37). It is important to note that the present study focused primarily on the hippocampal CA1 region. Given the well-established roles of other brain areas, such as the prefrontal cortex and amygdala, in METH-induced neuropsychiatric deficits, future studies will be essential to determine whether the beneficial effects of EA and the underlying mechanisms identified here extend to these regions.

Neuroinflammation is a central pathogenic mechanism in METH-induced neurotoxicity (38). Our study demonstrated that METH exposure induced robust activation of microglia, as evidenced by morphological alterations and enhanced expression of Iba-1. Concurrently, WB analysis revealed elevated levels of pro-inflammatory cytokines IL-6 and TNF-*α*, as well as activation of the NF-κB/NLRP3 signaling axis. These inflammatory changes were significantly suppressed by low-frequency EA treatment. This suggests that low-frequency EA may exert its beneficial effects by dampening neuroinflammatory responses and modulating microglial reactivity. Previous studies have demonstrated that EA can inhibit the NF-κB pathway and reduce inflammasome activation in various models of CNS injury, supporting our current observations (39–41).

Furthermore, an important aspect of our findings is the frequency-dependent effect of EA. While both 2 Hz and 10 Hz stimulation were applied at the same acupoint, only low-frequency EA produced significant neurobehavioral and neuroprotective outcomes, high-frequency EA showed no significant therapeutic effect. Studies have shown that low-frequency EA stimulation at ST36 activates the vagus-adrenal network via Prokr2-expressing neurons at the acupoint, thereby engaging the parasympathetic nervous system (42). This activation leads to the release of catecholamines, exerting anti-inflammatory effects (43). In contrast, high-frequency EA at ST36 has been found to stimulate the sympathetic nervous system, reducing stress resistance. In conclusion, the distinct mechanisms of low- and high-frequency EA interventions may be attributed to the natural rhythm of low-frequency EA, which likely activates the endorphin system, whereas high-frequency EA directly modulates neural conduction pathways through rapid stimulation. These differential mechanisms of frequency-specific EA modulation in the context of METH-induced pathology warrant further investigation.

Our study highlights the potential of low-frequency EA as a promising non-pharmacological intervention for METH-induced neuropsychiatric disorders. Previous studies have demonstrated that high-frequency EA effectively alleviates opioid-induced inflammatory pain, likely through the activation of peripheral *κ*-opioid receptors by EA stimulation (44). But this is the first study to systematically investigate the effects of conventional EA at ST36 on METH-induced depressive-like behaviors and cognitive impairment, and to elucidate its underlying mechanisms involving BBB integrity, neuroinflammation, and neuronal survival. These findings provide new insights into the integration of traditional Chinese medicine approaches with modern neuropsychiatric treatment strategies.

Nevertheless, several limitations should be acknowledged. The study utilized an acute METH exposure model, which may not fully recapitulate the chronic neuroadaptations seen in human addiction. Furthermore, our investigation focused on the hippocampal CA1 region, while other brain regions such as the prefrontal cortex and amygdala also play critical roles in emotion and cognition. This study aims to evaluate the therapeutic potential of EA in mitigating METH-induced neurobehavioral impairments and to elucidate the underlying molecular mechanisms. Our findings suggest that suppression of the NF-κB/NLRP3 signaling axis may represent a key mechanism through which EA exerts neuroprotective effects. Although our data indicate that low-frequency EA alleviates neuroinflammation via the NF-κB/NLRP3 pathway, we acknowledge that a causal relationship has not been definitively established. The absence of pathway-specific inhibition or genetic manipulation experiments precludes a conclusive assertion that the effects of EA are entirely mediated through this pathway. Nonetheless, our results are highly consistent with independent studies employing widely used NF-κB/NLRP3 inhibitors (45, 46). These findings have been further corroborated using other pharmacological inhibitors, such as ACT001 and JC124. By adopting comparable methodological approaches, these studies demonstrated that inhibition of the NF-κB/NLRP3 pathway significantly downregulates microglial activation markers, reduces the production of M1 polarization-related factors, and ameliorates inflammation-associated behavioral phenotypes and histopathological damage (47, 48). These results functionally establish a causal link between specific inhibition of this pathway and the induction of a quiescent microglial state.

Notably, this causality has been consistently replicated across multiple independently established experimental models, underscoring not only the reproducibility of the findings but also the specificity and generalizability of the mechanism. These observations support the biological plausibility of the proposed pathway. Future studies employing targeted interventional experiments are warranted to confirm the causal role of this signaling axis in EA-mediated neuroprotection.

This study has several limitations. All experiments were conducted exclusively in male mice. Given well-established sex differences in METH metabolism, neurotoxicity, and immune responses, the generalizability of our findings to females remains limited. Future studies—which we have already planned—will be necessary to include both sexes, in order to determine whether the protective effects of EA against METH-induced functional deficits are sex-specific or conserved. Finally, the therapeutic timeline in this study was relatively short. While we demonstrated that a 7-day EA intervention effectively alleviated METH-induced deficits, it remains unknown whether these benefits are sustained long-term after treatment cessation. The transient or durable nature of these effects is a critical factor for clinical relevance and will be a primary focus of our future investigations, which will include long-term survival studies with delayed behavioral and molecular analyses. While our data demonstrate that EA significantly reduces immobility in the TST and FST—behaviors often interpreted as despair-like states—it is important to acknowledge that these tests capture only certain dimensions of depression-related behavior, such as passive stress coping. They do not fully recapitulate the complex ethology of human depressive disorders, which include additional features such as anhedonia, appetite changes, and social withdrawal. Thus, our conclusions are necessarily constrained to these behavioral despair paradigms, and further studies employing additional models (e.g., sucrose preference for anhedonia, social interaction tests) are warranted to more comprehensively evaluate the antidepressant-like potential of EA.

## 5 Conclusion

Our findings revealed the potential of low-frequency (2 Hz) EA at ST36 acupoint in attenuating depressive-like behaviors and cognitive impairment caused by METH abuse. Additionally, EA intervention reduced METH-induced damage to the BBB and neuronal injury. These effects appear to be decreased by the attenuation of hippocampal microglial activation, decreased pro-inflammatory cytokines (including IL-6 and TNF-α), and the suppression of the NF-κB/NLRP3 signaling pathway. Taken together, low-frequency EA has the therapeutic potential as a non-pharmacological strategy for the treatment of METH-related neuropsychiatric disorders. Future studies will further investigate these findings in chronic METH exposure models and offer a solid theoretical foundation for its clinical applications.

## Data Availability

The original contributions presented in the study are included in the article, further inquiries can be directed to the corresponding authors.
