# Supplementary figures and images for: Low-frequency electroacupuncture attenuates methamphetamine-induced depressive-like behaviors and cognitive impairment via modulating neuroinflammation
Source: Front Neurol. 2025 Oct 20;16:1652065. doi: 10.3389/fneur.2025.1652065 (PMC12580150; doi:10.3389/fneur.2025.1652065)

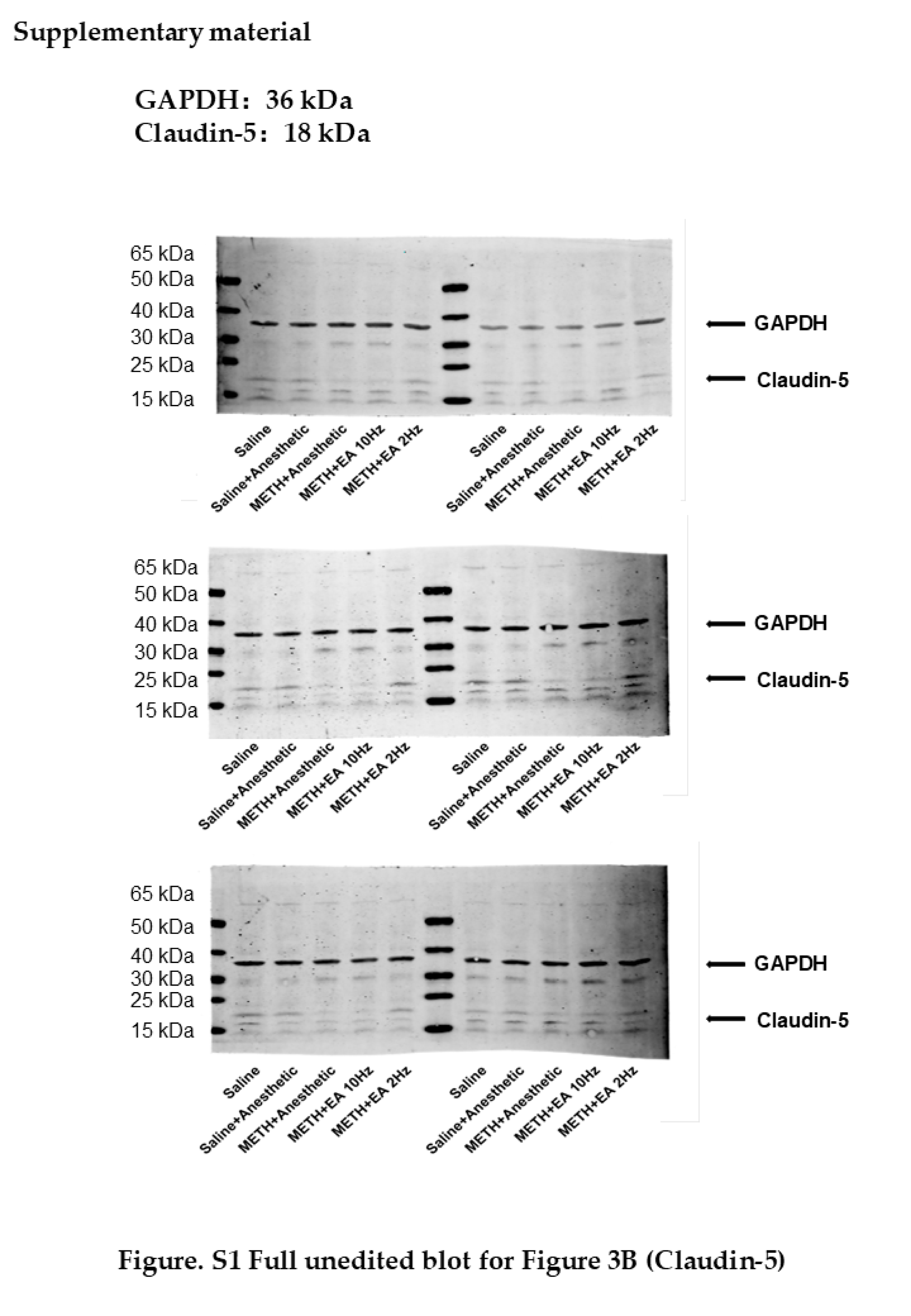

Supplement: Supplementary file 1 [file Image_1.tif]

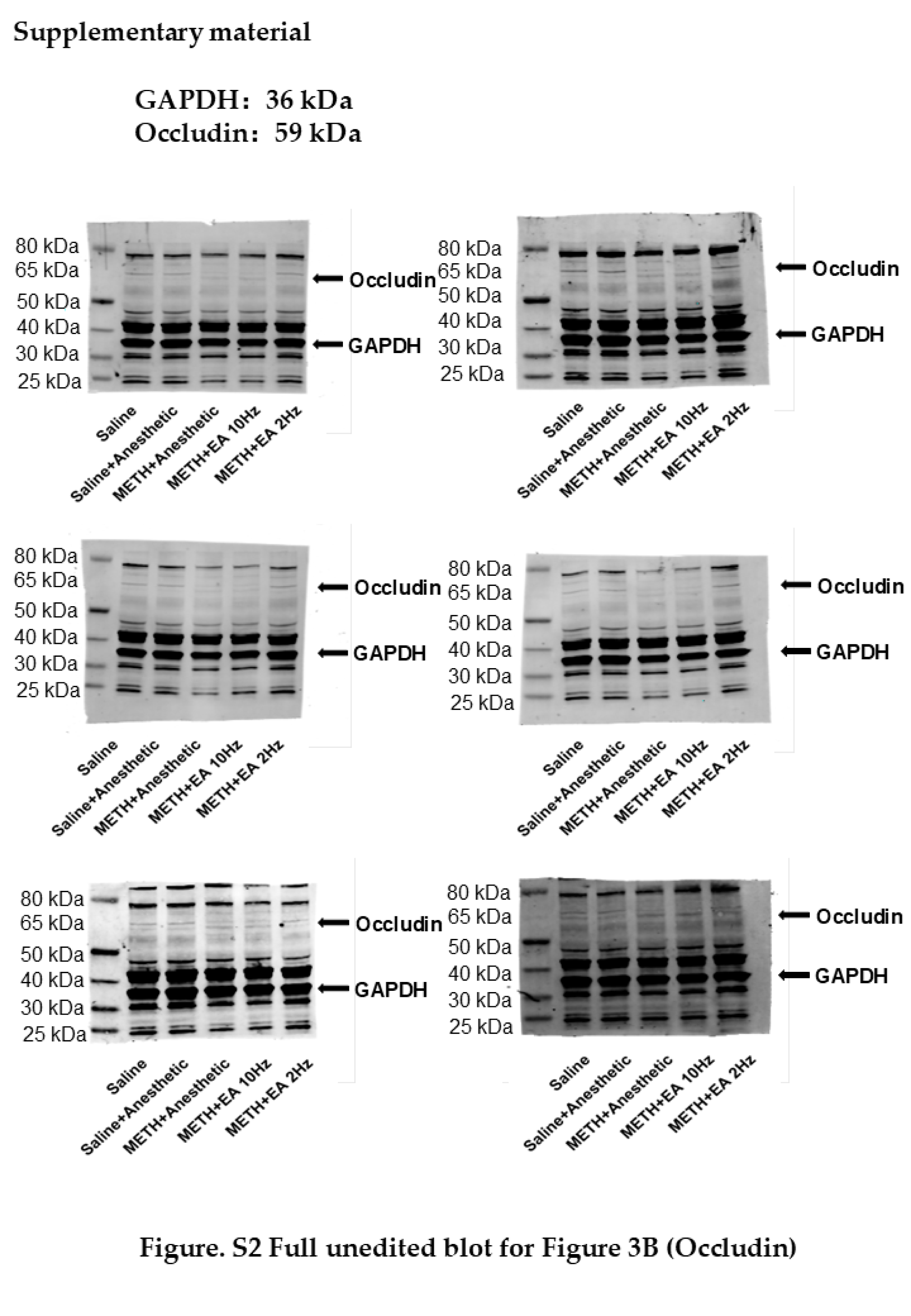

Supplement: Supplementary file 2 [file Image_2.tif]

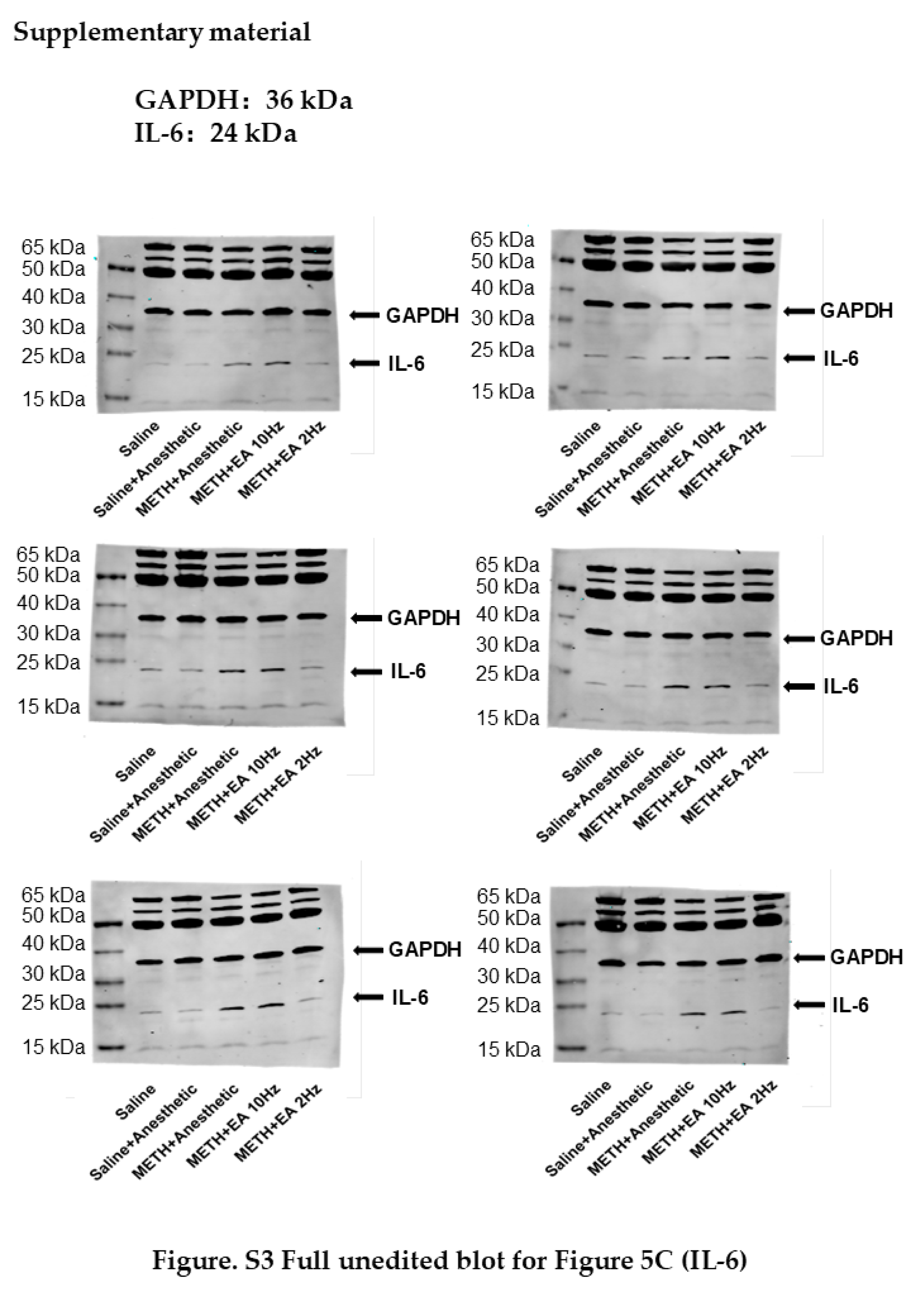

Supplement: Supplementary file 3 [file Image_3.tif]

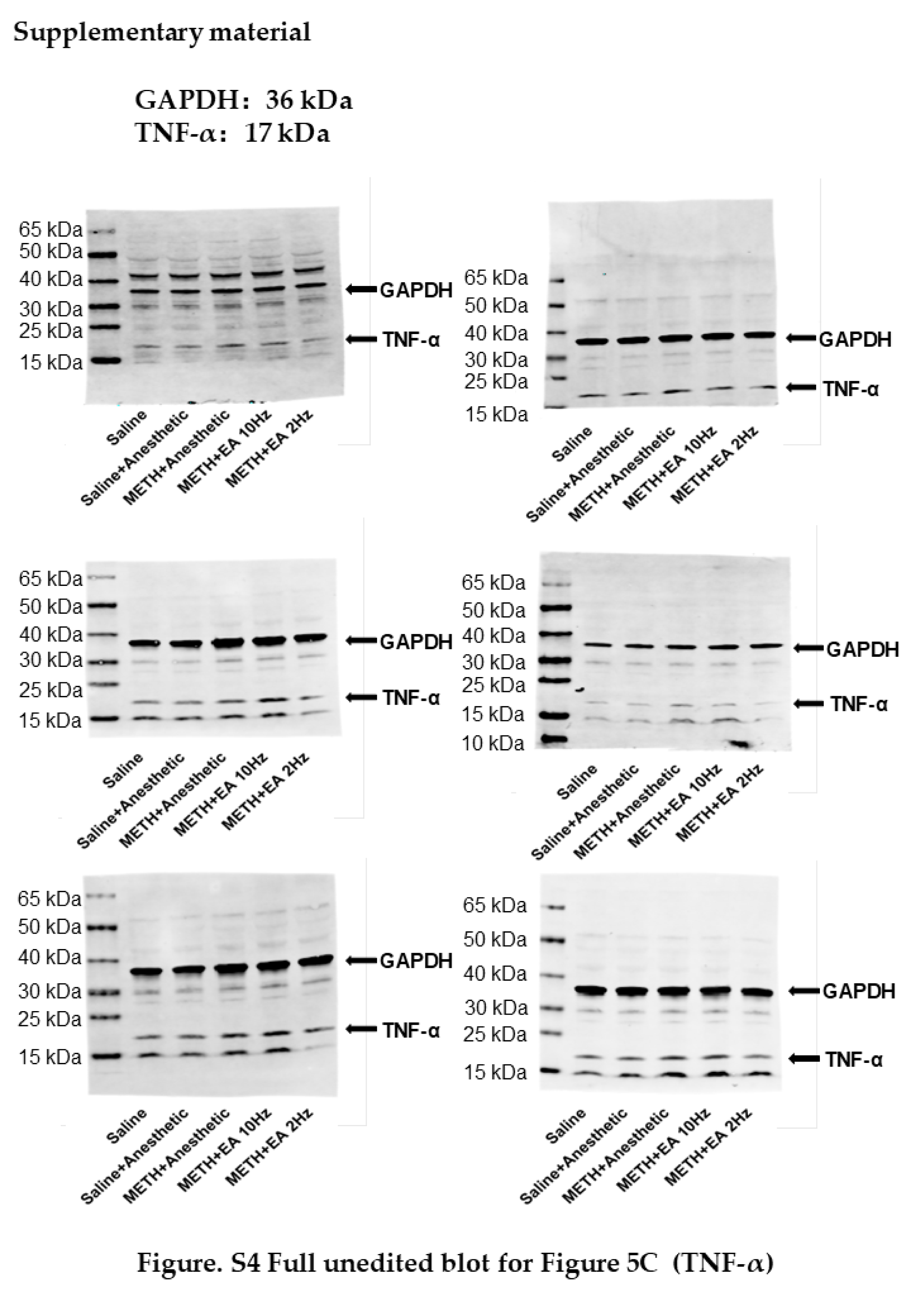

Supplement: Supplementary file 4 [file Image_4.tif]

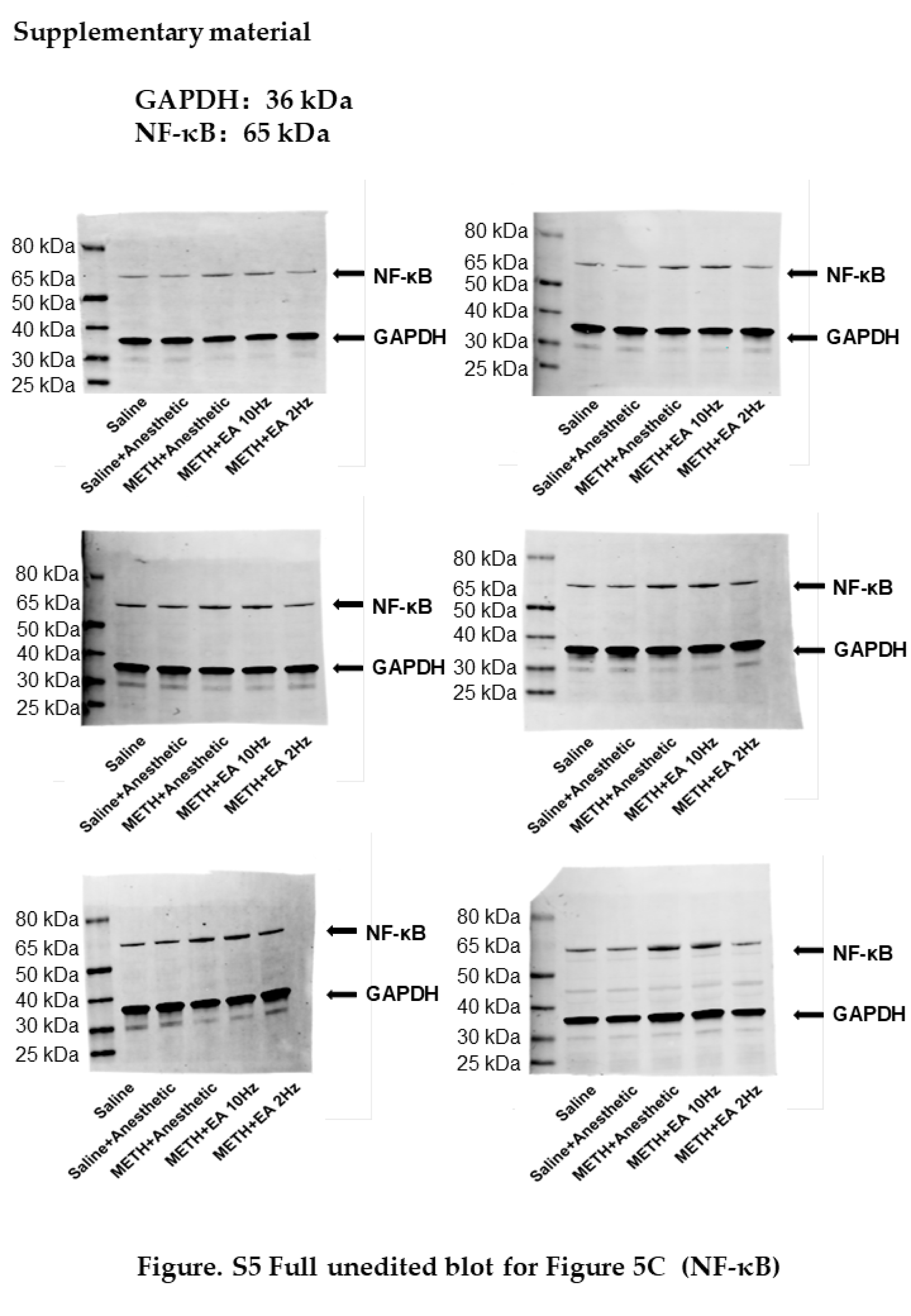

Supplement: Supplementary file 5 [file Image_5.tif]

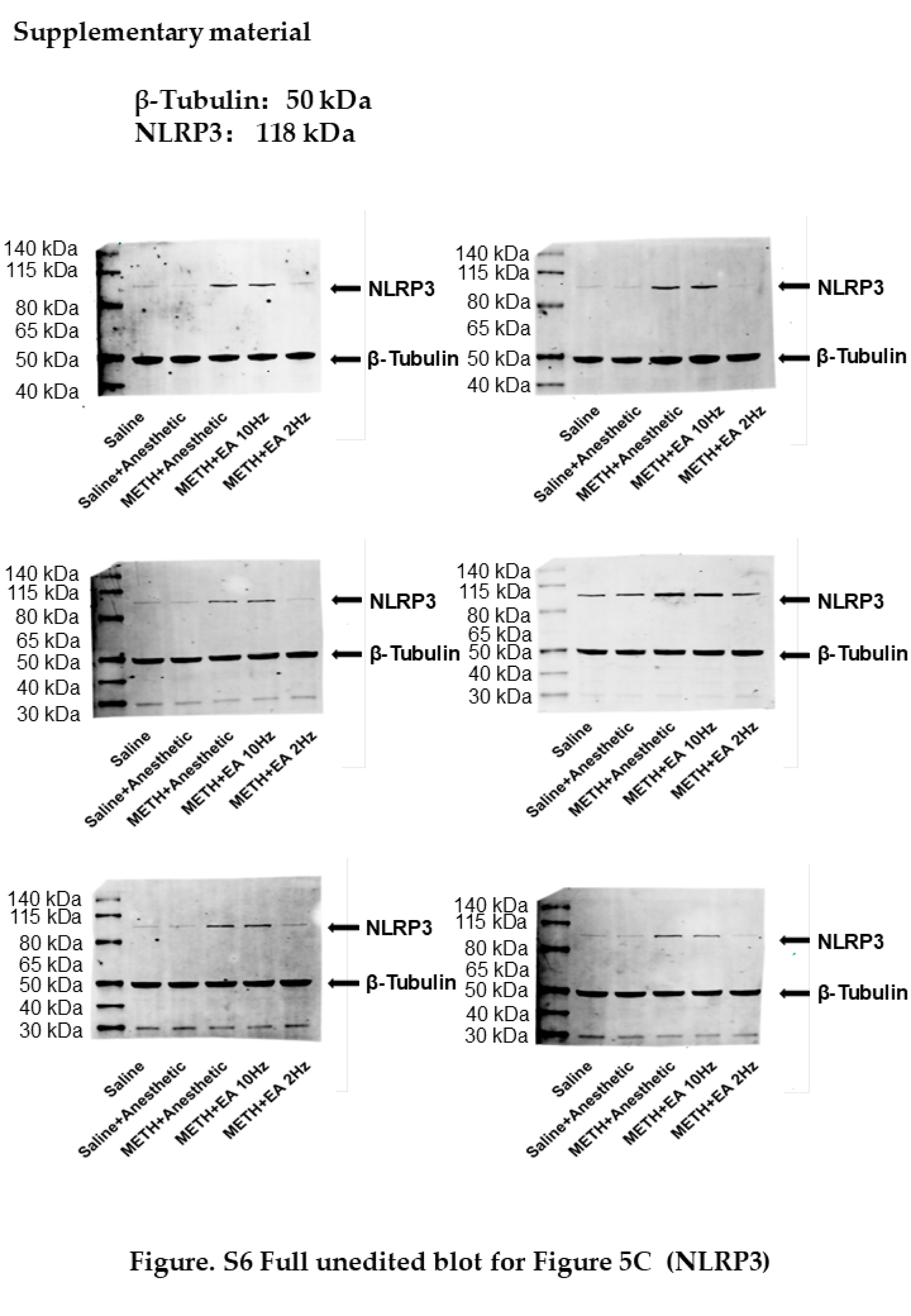

Supplement: Supplementary file 6 [file Image_6.tif]
